# Effects of *Lycium barbarum* Polysaccharide on Endoplasmic Reticulum Stress and Oxidative Stress in Obese Mice

**DOI:** 10.3389/fphar.2020.00742

**Published:** 2020-05-26

**Authors:** Feng-Lian Yang, Yu-Xia Wei, Bi-Yun Liao, Gui-Jiang Wei, Hai-Mei Qin, Xiao-Xia Pang, Jun-Li Wang

**Affiliations:** ^1^ Youjiang Medical College for Nationalities, Baise, China; ^2^ Reproductive Center, Affiliated Hospital of Youjiang Medical College for Nationalities, Baise, China

**Keywords:** *Lycium barbarum* polysaccharide, infertility, obesity, endoplasmic stress, oxidative stress

## Abstract

**Background:**

The incidence of obesity-associated decline in male fertility has increased over the years. *Lycium barbarum* polysaccharide (LBP), a natural plant polysaccharide extracted from the Chinese herb *L. barbarum* has shown promising therapeutic effects in overcoming the same.

**Aim:**

This study aimed to investigate the protective effect of LBP on the testes of obese mice.

**Methods:**

Following administration of LBP to high-fat diet-induced obese mice for 35 days, serum, sperm, and testis samples were obtained for subsequent experiments. Biochemical analysis and sex hormone content determination were performed to observe changes in glycolipid metabolism and testosterone levels, respectively, in the blood. Hematoxylin and eosin staining were carried out to assess the pathological changes in the testicular tissue. Oxidative stress levels were detected using enzyme-linked immunosorbent assay and expression levels of endoplasmic reticulum stress markers were determined using western blot in the testicular tissue.

**Results:**

Our results suggested that LBP reduced glucose levels and insulin resistance, increased testosterone levels and insulin sensitivity, and decreased testicular oxidative stress and pathological damage in obese mice. In addition, LBP down-regulated the expression of p-eIF2α, GRP78, and CHOP in the testicular tissues of obese mice.

**Conclusion:**

Our results show that LBP is a potential novel drug for preventing male infertility caused by obesity.

## Introduction

Obesity not only increases the risk of hypertension, diabetes, and cardiovascular disease (Di Vincenzo et al., 2018), but its impact on the reproductive system is also being increasingly valued (Kahn and Brannigan, 2017; Liu and Ding, 2017). Declining fertility is one of the most important and common complications in obese men and approximately 90% of the obese patients have varying degrees of reproductive dysfunction (Abdel-Fadeil et al., 2019; Crean and Senior, 2019). Obesity can cause and aggravate male factor infertility through endocrine abnormalities, hormone levels, semen parameters, sperm DNA integrity, and related comorbidities (Yan et al., 2015; Craig et al., 2017; Ferigolo et al., 2019). Studies have found that obesity not only has a negative effect on the patient's own fertility, but can even disrupt the early embryonic cell cycle dynamics, thereby reducing the fertility of the offspring (Deshpande et al., 2019; Raad et al., 2019).

The endoplasmic reticulum (ER) is a location for intracellular protein synthesis, folding, post-translational modifications, and maintaining the dynamic balance of Ca^2+^ (Cui et al., 2017). ER stress (ERS) is closely related to obesity-induced metabolic dysfunction, including testicular reproductive dysfunction (Martin-Jiménez et al., 2017; Park et al., 2017). Studies have shown that ERS injury is one of the main causes of abnormal spermatogenesis and persistent excessive ERS can interfere with the testicular germ cell-mediated secretion of related proteins or cytokines, thereby affecting testicular spermatogenesis (Guo et al., 2015). Studies have shown that whenever reactive oxygen species (ROS) release exceeds the capacity of endogenous antioxidants, oxidative stress occurs, which is inextricably linked to obesity and related diseases (especially insulin resistance and type 2 diabetes) (Niemann et al., 2017). In addition, elevated ROS levels are considered to be a risk factor for approximately half of male infertility cases among men diagnosed with sperm dysfunction (Kumar and Singh, 2018). Excessive ROS production is considered to be an important underlying mechanism for obese men's reproductive decline (Abbasihormozi et al., 2019). ROS and oxidative stress will destroy the ER function (Wang et al., 2020). Interestingly, a large amount of ROS will also be produced during the ERS process, further leading to oxidative stress (Zeeshan et al., 2016). The increase in antioxidants such as glutathione and superoxide dismutase can eliminate ROS and further improve oxidative stress and ERS (Liu et al., 2015). In conclusion, ERS and oxidative stress plays a crucial role in testicular germ cell damage and may be the main target for delaying testicular hypofunction. Given that obesity plays such an important role in the pathogenesis of male fertility, the need for developing drugs to improve the fertility of obese patients is imminent.

*Lycium barbarum* (*L. barbarum*) is a traditional Chinese medicine commonly used to treat infertility in China (Shi et al., 2017b). *Lycium barbarum* polysaccharide (LBP), a natural plant polysaccharide extracted from *L. barbarum*, has been shown to have a wide range of pharmacological effects, including anti-oxidation, anti-aging, immune regulation, and reproductive protection (Shi et al., 2017a; Yang et al., 2018). However, the effects of LBP on ERS in the testes of obese mice and their underlying mechanisms remain poorly understood. Therefore, the aim of this study was to determine the effects of LBP on glycolipid metabolism, sperm parameters, testicular oxidative stress, and ERS-related factors in obese mice and to unravel the mechanisms and functions of LBP in obesity and obesity-related male fertility.

## Methods and Materials

### Animal and Diets

This experiment was conducted in line with the animal ethics committee of Youjiang Medical University for Nationalities. Eight-week-old C57BL/6J male mice were purchased from Guangdong Medical Laboratory Animal Center (Foshan, China). All the animals were housed in an environment of 12 h light and 12 h dark cycle, in a temperature (24 ± 2°C) and relative humidity 45 ± 10%. They were randomized by body weight and fed a standard chow (10% kcal as fat; normal group) or a high-fat diet (HFD) (65% kcal as fat) provided by Beijing HFK Bioscience Co., Ltd. (Beijing, China) for 4 weeks. After 4 weeks, mice fed HFD weighed 20% more than normal-fed mice were the criteria for the successful construction of the obesity model.

### Experimental Design

Obese mice were randomly divided into three groups based on the bodyweight: obese model group (n = 9), 20 mg/kg (n = 9), and 40 mg/kg LBP-treated groups (n = 9). LBP was supplied by Fuzhou Teng Yuan Biological Technology Co., Ltd. (107-43-7, Fuzhou, China), which is derived from Ningxia wolfberry (fruit), and meets various national quality testing requirements (including various physical and chemical properties, pesticides, heavy metals, microorganisms, etc.), and the LBP content is 90.3% (national standard >90.0%). These groups were administered LBP or vehicle (equal amount of saline) by gavage for 5 weeks. Bodyweight, food intake, and blood glucose were recorded weekly of mice for all groups. After 5 weeks of administration, all mice were sacrificed by cervical dislocation, and the serum, testis, and epididymis were taken out, stored at −80°C or fixed in 10% formalin for subsequent experiment.

### Oral Glucose Tolerance Test (OGTT)

The glucose tolerance of each group mice was measured during the fifth week of administration. Mice have fasted for 12 h and glucose was administered by gavage at a dose of 2 g/kg. Glucose levels in blood from mouse tail were measured at 0, 30, 60, and 120 min using a glucose meter (Johnson & Johnson Medical, Shanghai, China), and glucose tolerance was assessed by calculating the area under the curve (AUC) of glucose.

### Biochemical and Sex Hormones Analysis

Fasting blood glucose (FBG) levels of mice were monitored by glucometer (Accuchek, Roche) weekly. Serum levels of low-density lipoprotein cholesterol (LDL-C), high-density lipoprotein cholesterol (HDL-C), triglycerides (TG), total cholesterol (TC), and testosterone (T) were measured by enzyme-linked immunosorbent assay (ELISA) kits (Nanjing, China) according to the manufacturer's instructions.

### Hematoxylin and Eosin (HE) Staining and Spermatozoa Analysis

First, we used HE staining to evaluate the pathological changes of testicular tissue. Mice were sacrificed by cervical dislocation, and the testes were quickly removed, immediately fixed in 10% formalin (Yi-li Fine Chemical, Beijing, China). Then embedded in paraffin following the normal procedure. Sections of 4-μm thicknesses were used for HE staining, which was performed in line with prior methods (An et al., 2017). Ultimately, the morphological changes of the testicular tissue sections were observed using an optical microscope (Olympus, Tokyo, Japan). In addition, semen was obtained from the tail of the epididymis and transferred to M2 medium (SIGMA, M7167) and incubated in a 37°C water bath for 15 min. Samples were then analyzed immediately using a computer-assisted semen analyzer (TOXIVOS II; Hamilton Thorne, Beverly, MA) to evaluate sperm count, viability, motility, morphology, and density parameters.

### Detection of Indicators Related to Oxidative Stress

All testicular samples should be tested on the same day after homogenization to avoid repeated freezing and thawing to reduce enzyme activity. If the sample protein concentration is too high, dilute the appropriate concentration if necessary, and then perform subsequent biochemical indicators. According to the steps indicated in the manual, the MDA (malondialdehyde), SOD (Superoxide dismutase), and GSH (glutathione) indexes in the testicular tissue were detected. The kit was purchased from the Nanjing Institute of Biology.

### Western Blot Analysis

Protein was extracted from testicular tissue using the Whole Protein Extraction Kit (Roche, Shanghai, China) and protein concentration was determined using the BCA Protein Assay Kit (Pierce Biotechnology, Rockford, IL, USA). The protein sample was separated on 10% of the SDS-PAGE gel and then transferred to PVDF membranes. After being blocked with 5% non-fat milk, the membranes were incubated overnight with primary antibodies against C/EBP homology protein (CHOP, 1:1,000), activating transcription factor 4 (ATF4, 1:1,000), glucose regulated protein 78 (GRP78, 1:1,000), phosphorylated eukaryotic initiation factor-2 alpha (p-eIF2α, 1:1,000), or glyceraldehyde-3-phosphate dehydrogenase (GAPDH, 1:2,000) overnight at 4°C, then incubated with the secondary antibody (1:5,000). Rabbit monoclonal phospho-eIF2α antibody was purchased from Cell Signaling Technology (Danvers, MA, USA). Rabbit monoclonal GRP78 and CHOP antibodies were obtained from Abcam (Cambridge, MA, USA). Protein bands were visualized using enhanced chemiluminescence and quantified by densitometry scanning using Quantity One (Bio-Rad Laboratories, Hercules, CA, USA), and GAPDH was used as the loading control.

### Statistical Analysis

Data were analyzed using Statistical Package for Social Sciences (SPSS) software version 17 and expressed as mean ± standard error of mean (SEM). Multiple group comparisons were determined by one-way analysis of variance (ANOVA) followed by Duncan's analysis. *P* < 0.05 were considered significant.

## Results

### LBP Decreases Body Weight and Food Intake in Obese Mice

**Figure 1:**
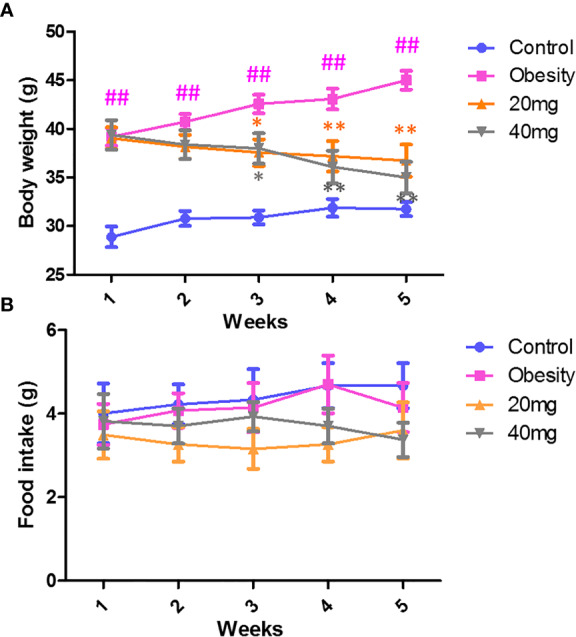
Effect of LBP on **(A)** body weight and **(B)** food intake in obese mice. Values are expressed as mean ± SEM (n = 9). *^##^P < 0.01, versus* control group; **P < 0.01, * *P < 0.05 versus* obesity model group.

As shown in **Figure 1A**, during the treatment period, the body weights of the obese mice were always found to be greater than those of the control group. From the third week of LBP intervention, mice in the 20 and 40 mg intervention groups showed a trend of weight loss compared to the obesity model group (P < 0.05). After 4 weeks of intervention, the body weights of mice in the 20 and 40 mg intervention groups were significantly reduced compared to the obesity model group (P < 0.01). In addition, our data showed that during the intervention, there was no difference (P > 0.05) in the food intake between the different groups of mice (**Figure 1B**).

### Effects of LBP on FBG and OGTT in Mice With Obesity

**Figure 2:**
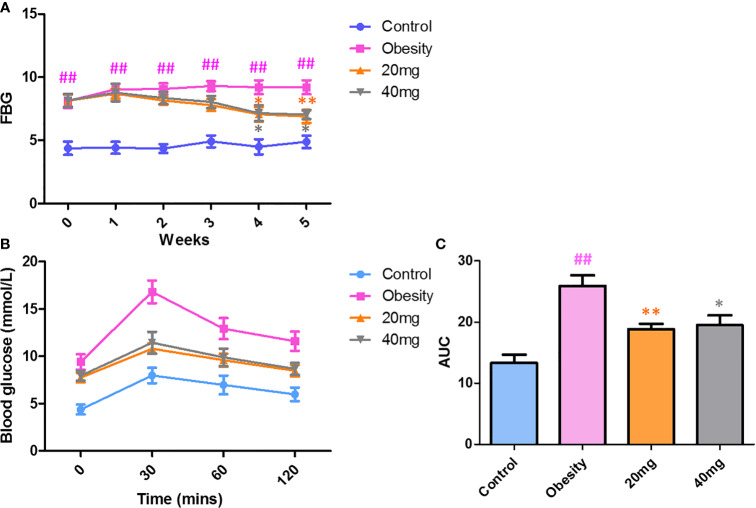
Effect of LBP on FBG **(A)** and OGTT **(B, C)** levels in obese mice. Values are expressed as mean ± SEM (n = 9). *^##^P < 0.01* *versus* control group; **P < 0.01, *P < 0.05 versus obesity model group. FBG, fasting blood glucose; OGTT, oral glucose tolerance test; AUC, area under the curve.

As shown in **Figure 2**, before LBP administration, the fasting blood glucose (FBG) levels and AUC of the obese mice were found to be significantly higher (P < 0.01) from those of the control mice, indicating that the high-fat diet (HFD)-induced obese insulin resistance mouse model was successful. After 4 weeks of intervention, mice treated with 20 and 40 mg LBP displayed significantly lower (P < 0.05) FBG levels as compared to those in the obese group. In addition, the OGTT trial showed that mice in the 20 and 40 mg LBP intervention groups had significantly lower glucose levels (P < 0.05) at 0, 30, 60, and 120 min of glucose intervention, as compared to the obese group. These results indicate that LBP can reduce FBG levels and improve insulin sensitivity in obese mice.

### Effects of LBP on Serum Lipid Profiles

**Figure 3:**
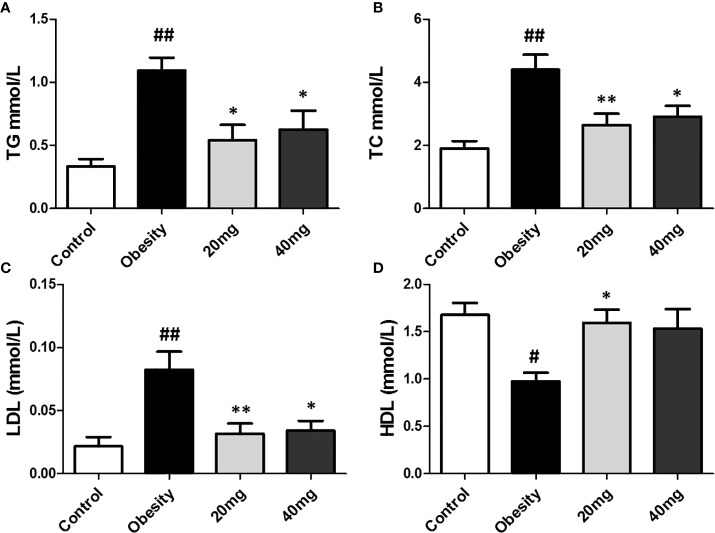
Effect of LBP on serum lipid profile in obese mice. **(A)** triglyceride; **(B)** total cholesterol; **(C)** low density lipoprotein cholesterol; **(D)** high density lipoprotein cholesterol. Values are expressed as mean ± SEM (n = 9). *^##^P < 0.01*, ^#^ *P < 0.05 versus* control group; **P < 0.01, *P < 0.05 versus obesity model group. TG, triglyceride; TC, total cholesterol; LDL, low density lipoprotein cholesterol; HDL, high density lipoprotein cholesterol.

As shown in **Figure 3**, compared to the control group, the serum TC, TG, and LDL-C levels were significantly higher (P < 0.01), while the serum HDL-C levels were significantly lower (P < 0.01) in the HFD-induced obesity group. After 5 weeks of LBP treatment, as compared to the obese group, the serum TC, TG, and LDL-C levels were significantly lower (P < 0.05), while the serum HDL-C levels were significantly higher in the treated mice. This suggests that LBP can improve lipid metabolism in obese mice.

### Effects of LBP on Oxidative Stress in the Testes of Obese Mice

**Figure 4:**
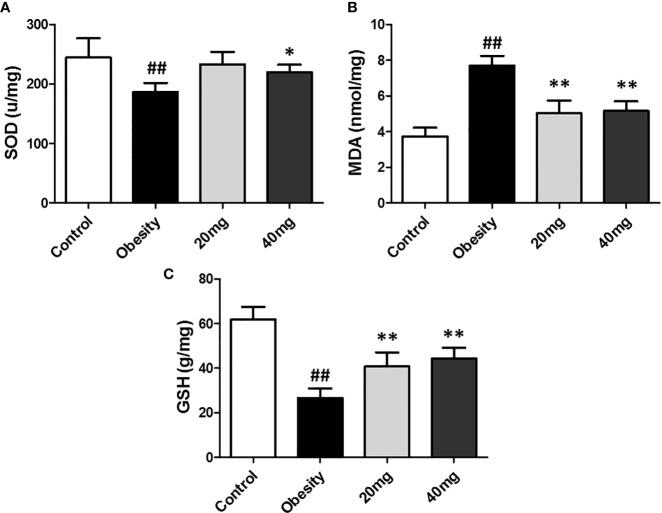
Effect of LBP on the expression levels of MDA, SOD, and GSH in the testis of obese mice. **(A)** SOD, **(B)** MDA, and **(C)** GSH. Values are expressed as mean ± SEM (n = 9). *^##^P < 0.01*, *versus* control group; **P < 0.01, *P < 0.05 versus obesity model group. MDA, malondialdehyde; GSH, glutathione; SOD, Superoxide Dismutase.

As shown in **Figure 4**, the expression levels of MDA was significantly higher (P < 0.01), while the expression levels of SOD and GSH were significantly lower (P < 0.05) in the testis tissues of the obese model group, as compared to the control group. Both 20 and 40 mg LBP interventions resulted in an increase in the expression levels of SOD and GSH and a decrease in the expression level of MDA in the testis of the treated mice, compared to the obese mice (P < 0.05). These results showed that LBP could regulate the expression levels of the anti-oxidant molecules, SOD, GSH, and MDA in the testes of obese mice.

### Effects of LBP on Sex Hormones in Mice With Obesity

**Figure 5:**
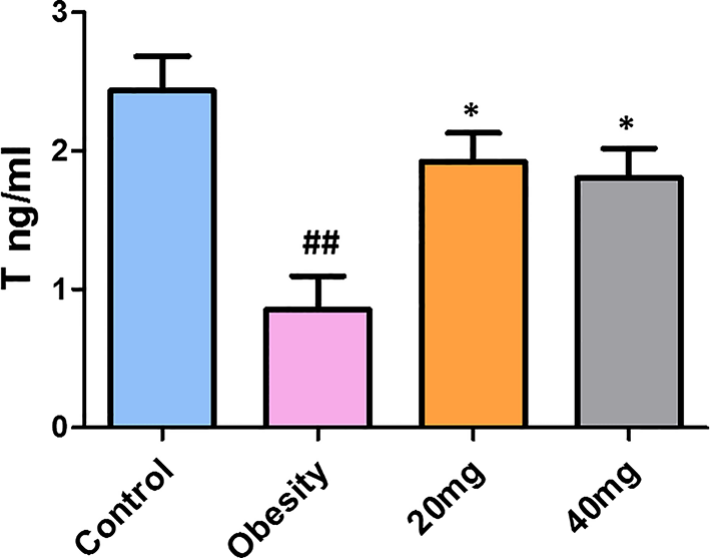
LBP can increase serum testosterone levels in obese mice. Values are expressed as mean ± SEM (n = 9). *^##^P < 0.01*, *versus* control group; *P < 0.05, *versus* obesity model group. T, testosterone.

As shown in **Figure 5**, testosterone levels were significantly decreased in obese mice compared to control mice (P < 0.01). Treatment with both 20 and 40 mg of LBP significantly increased (P < 0.05) the serum testosterone levels of the treated mice, in comparison with the obese mice. Therefore, we suggest that LBP intervention can alleviate the damage caused by obesity in male mice by regulating the sex hormone levels.

### Effects of LBP on Sperm Vitality and Motility in Obese Mice

**Figure 6:**
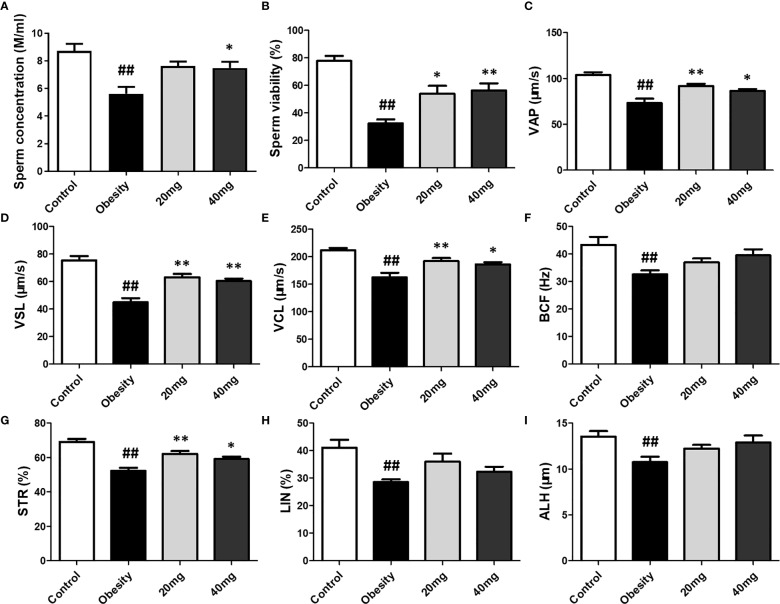
Effect of LBP on sperm vitality **(B)** and motility **(A)** in obese mice. Values are expressed as mean ± SEM (n = 9). *^##^P < 0.01*, *versus* control group; **P < 0.01, *P < 0.05 versus obesity model group. **(D)** VSL, straight-line velocity; **(C)** VAP, average path velocity; **(E)** VCL, curvilinear velocity; **(G)** STR, straightness; **(H)** LIN, linearity; **(F)** BCF, beat-cross frequency; **(I)** ALH, amplitude of lateral head displacement.

Compared to the control group, the sperm motility, concentration, VAP, VSL, VCL, BCF, STR, LIN, and ALH were significantly lower (P < 0.01) in the obese model group. Significantly higher (P < 0.05) sperm concentration was observed in the 40 mg LBP-treated mice, as compared to the obese mice. Although 20 mg LBP intervention also showed a trend toward higher sperm concentration in the treated mice, as compared to the obese mice, the effect was not statistically significant. In addition, 20 and 40 mg LBP-treated mice also displayed significantly higher (P < 0.05) sperm motility, VAP, VSL, VCL, BCF, and STR. Although the 20 and 40 mg LBP-treated groups also displayed higher BCF, LIN, and ALH levels compared to the obese model group, the differences were not statistically significant (P > 0.05). Thus, obesity led to a reduction in the sperm motility, concentration, and other sperm kinetic parameters. This reduction was rescued upon administration of LBP treatment to the obese mice (**Figure 6**).

### Protective Effect of LBP on the Testes of Obese Mice

**Figure 7:**
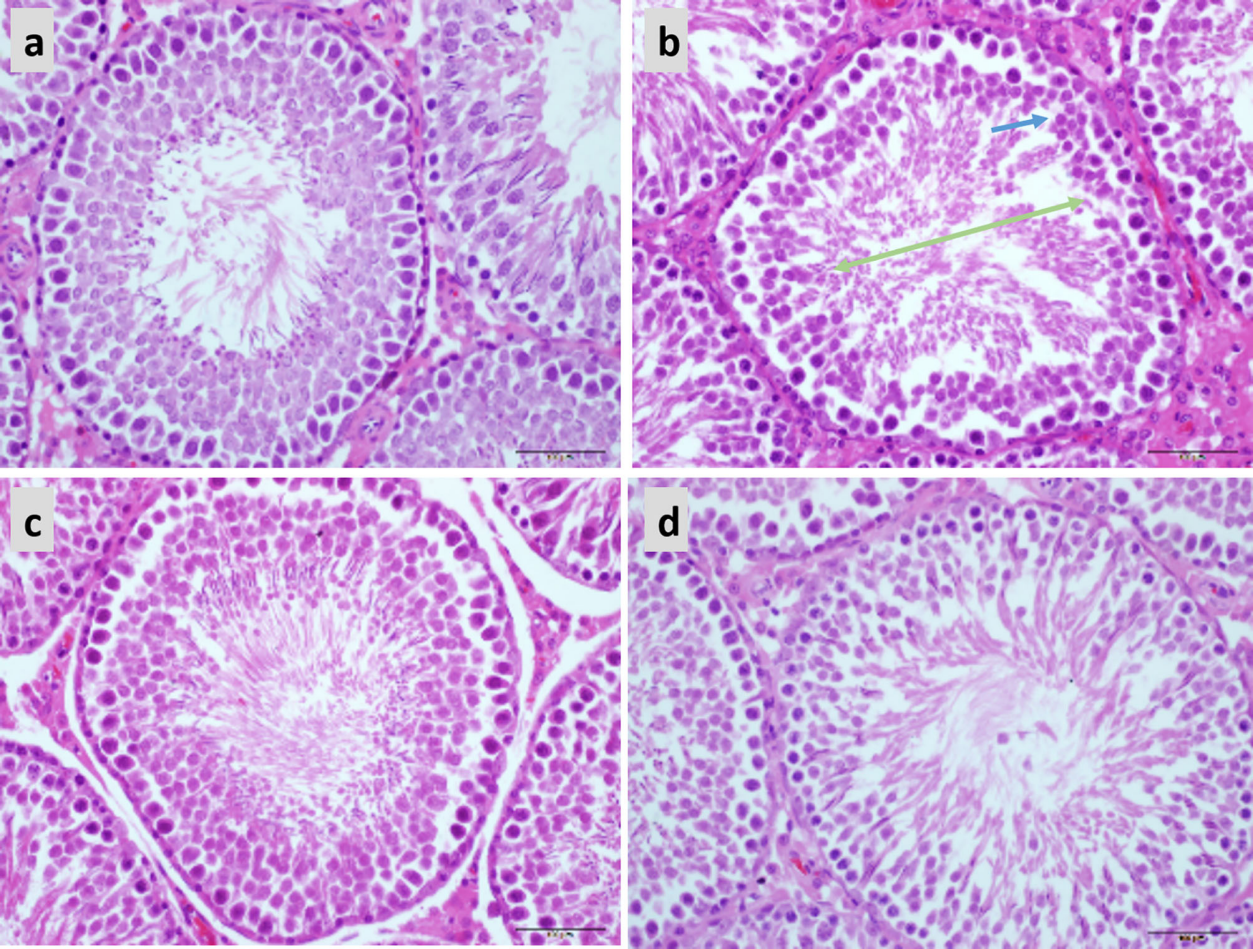
Hematoxylin and eosin staining showing the effect of LBP on testicular histology in obese mice (40X), Scale bar: 500 μm. **(A)** Testicular section of normal mice showed plenitudinous seminiferous tubules with large diameters and normal spermatogenic cells; **(B)** Testicular section of obese mice showed atrophic spermatogenic tubules with smaller diameters (Green arrow) and fewer spermatogenic cells (Blue arrow); **(C, D)** Testicular section of LBP administration shows the mouse as a spermatogenic tubule structure with normal diameter and shows spermatogenic cells in almost all spermatogenic tubules.

In the normal testicular tissue, the spermatocytes of the seminiferous tubules appear normal and are closely aligned. The cell structure is clear at all stages; it can be clearly observed that the Leydig cells between the seminiferous tubules are round and distributed in clusters and that there are a large number of mature sperm in the lumen (**Figure 7A**). Compared to the control group, the sperm count was decreased and the arrangement was disordered in the testicular tissue of obese mice. Sperm cells were clustered in the lumen, the spermatozoa appeared deformed, and the parenchymal cells were significantly reduced (**Figure 7B**). Treatment with both 20 and 40 mg LBP significantly improved the damaged testicular tissue (**Figures 7C–D**).

### Expression of Phosphorylated eIF2α, GRP78, and CHOP Proteins in the Testicular Tissue

**Figure 8:**
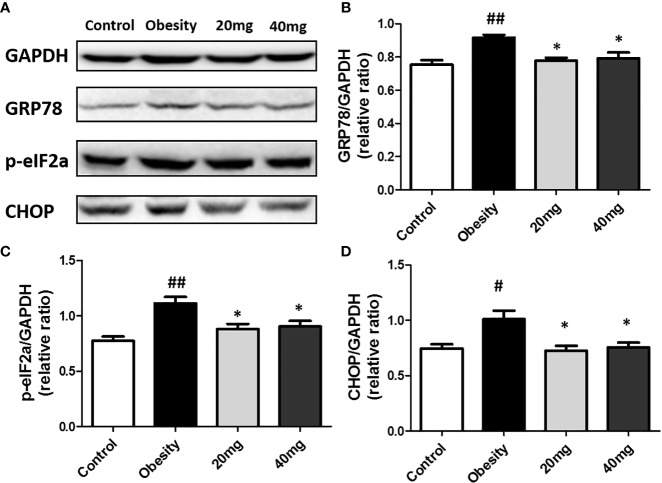
LBP can reduce the expression of p-eIF2a **(B)**, GRP78 **(C)**, and CHOP **(D)** proteins in obese mice **(A)**. Values are expressed as mean ± SEM (n = 9). *^##^P < 0.01*, ^#^ *P < 0.05 versus* control group; *P < 0.05 versus obesity model group.

The expression levels of ERS markers, such as p-eIF2α, GRP78, and CHOP in the testicular tissues were significantly higher (P < 0.05) in the obese mice compared to the control mice (**Figure 8**). After treatment with 20 and 40 mg LBP, the expression levels of p-eIF2α, GRP78, and CHOP in the treated mice were found to be lower (P < 0.05), as compared to the obese mouse model. Our results indicate that the expression levels of all ERS markers (including p-eIF2α, GRP78, and CHOP) were reduced in both the 20 and 40 mg intervention groups compared to the obesity model group. These results further illustrate that LBP can alleviate ERS in the testicular tissues of male obese mice.

## Discussion

In this study, we found that LBP supplementation could significantly improve the symptoms of HFD-induced obesity and its correlated indices as well as repair the impaired glucose tolerance in obese mice, thus showing an anti-obesity effect. These results are consistent with a previous report (Xiao et al., 2014). Abnormal lipid metabolism and weight gain are unavoidable in HFD-induced obese mice. Our results suggest that intervention using 20 and 40 mg LBP can reduce the serum TG, TC, and LDL-C levels, and increase the serum HDL-C levels, as previously described (Li et al., 2014). LBP showed a significant regulatory effect on blood glucose and lipid levels; both the LBP intervention groups showed regulation of glycolipid metabolism. In addition, our study also found that LBP intervention has a high potential to increase glucose tolerance. These results indicate that LBP treatment, at doses of 20 and 40 mg, has beneficial hypoglycemic and hypolipidemic effects.

Testis is an important reproductive organ in male animals with physiological functions, such as spermatogenesis and endocrine regulation. Numerous studies have demonstrated that HFD-induced obesity and insulin resistance affect spermatogenesis and maturation (Yan-Fei et al., 2018). In this study, compared to the control mice, the obese mice displayed significantly reduced number of sperms, sperm motility, and other sperm kinetic parameters in the epididymis and abnormal seminiferous tubule structures in the testicular tissue sections. However, the sperm parameters as well as the testicular tissue structure of the obese mice recovered to varying degrees upon LBP treatment, suggesting that LBP had evident spermatogenic properties. In addition, spermatocyte maturation and spermatogenesis are closely related to endocrine hormone levels. In this study, the serum levels of the endocrine hormone testosterone in the obese mice were significantly reduced compared to that in the control group; however, the serum testosterone levels were found to be significantly higher in the treated mice, following LBP intervention. In summary, LBP seems to be capable of significantly improving testicular dysfunction in obese mice, including spermatogenic dysfunction and endocrine disorders.

Oxidative stress is the main mechanism throughout the development of obesity and its complications (Koo and Kang, 2019). Previous studies have shown that all body reproductive damage caused by obesity is related to oxidative stress, including spermatogenic cell apoptosis, decreased spermatogenic ability, erectile dysfunction, and loss of libido (Leisegang et al., 2014; Škurla and Rybář, 2018). Under normal physiological conditions, ROS produced in the body can be dynamically balanced by the anti-oxidative stress defense system, including SOD, CAT, and GSH (Cannarella et al., 2019). Studies have found that obesity can affect the body's oxidative stress levels through a variety of ways, including energy metabolism and the mitochondrial electron transport chain, leading to increased ROS and male infertility (Leite et al., 2019). In the present study, compared to the control mice, expression levels of GSH and SOD were significantly decreased, while expression levels of MDA were significantly increased in the testis tissues of HFD-induced obese mice. Treatment with 20 and 40 mg LBP significantly increased the expression of SOD and GSH and decreased the expression of MDA in the obese mice. This study suggests that LBP has good anti-oxidant properties and its effect on improving testicular dysfunction may be mediated through anti-oxidation.

ERS plays an important role in the spermatogenic disorder of HFD-induced obese mice. SIRT1, with a decrease in ERS, and activation of silencing regulator 1, has been found in the testicular tissues of obese mice, which reveal that ERS stimulation is involved in the process of obesity-related male subfertility (Mu et al., 2018). The classical ERS pathway consists of three distinct branches: activation of transcription factor 6 (ATF6), protein kinase R-like endoplasmic reticulum kinase (PERK), and inositol-requiring enzyme 1 (IRE1) (Xu et al., 2015). The massive activation of GRP78 helps the protein fold to form the correct morphology and regulate the homeostasis of the ER (Li et al., 2015). Increased expression of GRP78 and CHOP proteins are two important markers of ERS (Kratochvílová et al., 2016). Activation of PERK can phosphorylate eIF2α and phosphorylation of eIF2α can reduce the transcription and translation of cells, thereby reducing protein production (Mo et al., 2019). Therefore, activation of the PERK/p-eIF2α pathway can reduce protein synthesis in the ER and regulate endoplasmic reticulum homeostasis to reduce stress in the ER. In the current study, the expression levels of GRP78, p-eIF2α, and CHOP were significantly higher in the testicular tissues of the obesity model group than that of the control group. After LBP intervention, the protein expression levels of GRP78, p-eIF2α, and CHOP were found to be significantly lower in the testicular tissues of the treated mice as compared to that in the obese mice, indicating that LBP could down-regulate the expression of GRP78, p-eIF2α, and CHOP, thus inhibiting ERS, and anti-male infertility caused by obesity.

The occurrence of ERS is closely related to oxidative stress (Karna et al., 2019a). When ERS occurs, the formation and breakage of disulfide bonds may generate ROS and gradually accumulate, eventually causing the occurrence of oxidative stress (Karna et al., 2019b). In addition, ERS can also cause mitochondrial dysfunction by increasing the production of ROS in mitochondria and interfere with the normal function of cells. Many studies have confirmed that ERS and oxidative stress can increase the imbalance of intracellular homeostasis through mutual positive feedback, and interfere with cell function and activate pro-apoptotic signals (Malhotra and Kaufman, 2007; Karna et al., 2019c). These conclusions suggest that a stable redox state is required during the folding of the ER protein, and ROS are also produced, stimulating the operation of the redox system in the cell. In addition, GSH is closely related to the state of cellular oxidative balance, is reductive and has the effect of improving oxidative stress and ERS (Yang et al., 2015). Our research shows that LBP intervention can improve ERS, oxidative stress, and increase the expression of antioxidant enzyme-related indicators in testis tissues of obese mice induced by HFD. This suggests that LBP can improve testicular function in male mice by regulating ERS and oxidative stress in the testis of obese mice.

In conclusion, LBP reduced glucose levels and insulin resistance, increased insulin sensitivity and testosterone levels, and inhibited oxidative stress damage as well as pathological damage in the testes of obese mice. LBP regulates the ERS pathway signaling by inhibiting activation of p-eIF2α and GRP78-CHOP. Our results suggest that LBP is a promising new drug to prevent male infertility caused by obesity. However, further research will be needed to understand the underlying mechanisms of LBP inhibiting ERS and oxidative stress and its effect on obesity-induced male infertility.

## Data Availability Statement

The raw data supporting the conclusions of this article will be made available by the authors, without undue reservation, to any qualified researcher.

## Ethics Statement

The study protocol was approved by the Animal Care and Management Committee of the Youjiang Medical University for Nationalities. All manipulations were at the request of the guidelines of the Animal Care Committee.

## Author Contributions

F-LY and J-LW designed the experiments. F-LY wrote the manuscript. F-LY, Y-XW, and B-YL performed the experiments. G-JW, H-MQ, and X-XP analyzed the data. All authors reviewed the manuscript.

## Funding

We would like to thank Dr Kehuan Lu for careful guidance. This study was supported by the Natural Science Foundation of Baise in China (No. 20182511), foundation of the Health Department of Guangxi Province, China (No. Z20170234; Z20170236), “139” medical high-level talent training plan and thousands of young and middle-aged backbone teachers cultivation plan of Guangxi Province, China, 2019 Natural Science Foundation of Guangxi in China (No. 2019GXNSFBA245034), and the Professional and experimental practice teaching base construction projects of Guangxi Province, China.

## Conflict of Interest

The authors declare that the research was conducted in the absence of any commercial or financial relationships that could be construed as a potential conflict of interest.

## Abbreviations

LBP, *Lycium barbarum* polysaccharide; ERS, endoplasmic reticulum stress; HFD, high fat diet; T, testosterone; FBG, fasting blood glucose; LDL-C, lipoprotein cholesterol; HDL-C, high density lipoprotein cholesterol; TG, triglycerides; TC, total cholesterol; OGTTs, oral glucose tolerance tests; VAP, average path velocity; VSL, straight-line velocity; VCL, curvilinear velocity; BCF, beat-cross frequency; STR, straightness; LIN, linearity; ALH, amplitude of lateral head displacement; GRP78, glucose regulated protein 78; eIF2α, eukaryotic initiation factor-2 alpha; ATF6, activating transcription factor 6; CHOP, C/EBP homology protein; AUC, area under curve; HE, hematoxylin eosin; ANOVA, analysis of variance; PERK, PKR-like ER kinase; IRE1, inositol-requiring enzyme 1; MDA, malondialdehyde; SOD, Superoxide dismutase; GSH, glutathione; CAT, catalase; GAPDH, glyceraldehyde-3-phosphate dehydrogenase.
